# A Wireless Emergency Telemedicine System for Patients Monitoring and Diagnosis

**DOI:** 10.1155/2014/380787

**Published:** 2014-05-06

**Authors:** M. Abo-Zahhad, Sabah M. Ahmed, O. Elnahas

**Affiliations:** Electrical and Electronic Engineering Department, Faculty of Engineering, Assiut University, Egypt

## Abstract

Recently, remote healthcare systems have received increasing attention in the last decade, explaining why intelligent systems with physiology signal monitoring for e-health care are an emerging area of development. Therefore, this study adopts a system which includes continuous collection and evaluation of multiple vital signs, long-term healthcare, and a cellular connection to a medical center in emergency case and it transfers all acquired raw data by the internet in normal case. The proposed system can continuously acquire four different physiological signs, for example, ECG, SpO2, temperature, and blood pressure and further relayed them to an intelligent data analysis scheme to diagnose abnormal pulses for exploring potential chronic diseases. The proposed system also has a friendly web-based interface for medical staff to observe immediate pulse signals for remote treatment. Once abnormal event happened or the request to real-time display vital signs is confirmed, all physiological signs will be immediately transmitted to remote medical server through both cellular networks and internet. Also data can be transmitted to a family member's mobile phone or doctor's phone through GPRS. A prototype of such system has been successfully developed and implemented, which will offer high standard of healthcare with a major reduction in cost for our society.

## 1. Introduction

**Figure 1:**
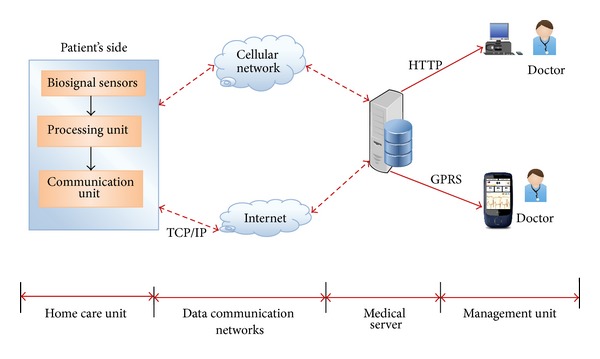
Main components of telemedicine system.

A healthcare system in the last decade was made possible due to the recent advances in wireless and network technologies, linked with recent advances in nanotechnologies and ubiquitous computing systems. The term telemedicine refers to the utilization of telecommunication technology for medical diagnosis, treatment, and patient care [1]. The aim of telemedicine is to provide expert-based healthcare to understaffed remote sites through modern telecommunication (wireless communications) and information technologies. One of the benefits of telemedicine is cost savings, because information is less expensive to transport than are people. Advances in medical technologies have led to accelerated growth of the elderly population in many countries, resulting in an increasing requirement for home health monitoring to ensure that elderly patients can lead independent lives [2]. Many physiological signals can be measured from individuals in their living environments during daily activities and are potentially applied to observe the deviations of health status in the early phase or to alert paramedics automatically in emergency cases [3]. Especially for remote monitoring of physiological parameters, all the studies developed and currently used in this area can be categorized by several aspects: type of sensors, type of data communication, monitoring device, and signal processing/medical algorithms [4]. So these aspects along with recent studies will be discussed in this section. As shown in Figure 1 the main telemedicine system components in recent years include biosignal sensors, processing units, data communication networks, and medical service center.

The biosignal sensors are responsible for acquiring the physiological data (patient's vital signs) and transmitting it to the signal processing unit. Several studies are made focusing only on designing these sensors to be tiny in size [5], maintain patient mobility [6], and consume low operating power to reduce battery size which can last for longer durations [7]. A collection of wearable medical sensors could communicate using personal area network or body network [8], which can be even integrated into user's clothes [9]. At the next stage, sensor layer of every remote monitoring system is typically connected to the processing device for signal acquisition, processing, analysis, and formatting data to be sent to the communication layer. The processing unit may evaluate patient status and trends in patient's medical condition. Processing unit can be PC [10], mobile phone [11], or embedded system (microcontroller, DSP processor, and FPGA) [12]. Many medical algorithms were developed in recent telemedicine studies to help in patient diagnosis [13] and early detection of cardiovascular diseases [14]. Among human vital signals, pulse assessment has long been a research area of interest in the physiology field, because the pulse reflects a person's state of health [15]. Many investigations have proposed monitoring systems that can measure various biosignals and provide QRS detection and arrhythmia classification [14], real-time ECG classification algorithm [13], and heart rate variability measurement [14]. Also recent advances in wireless and network technologies make it possible to develop a wireless telemedicine system which offers an effective means of bringing healthcare services to patients. Telemedicine systems can be divided into two modes of operations: real-time mode, in which patient data are available at the server end immediately after acquisition, and store-and-forward mode, which involves accessing the data at a later time. In both modes, the vital signs are transmitted via computer networks [16], cellular networks [17], public telephone networks [18], or cable TV networks [19] to the server. In these system models, an expert is expected at places where he/she can use a PC to access the server for analyzing the vital signs data, and the patient is bounded at a fixed place like home or healthcare center where a PC is equipped for transmitting these data. The use of wired network connected PCs limits the degree of freedom of both doctors and patients to move around.

To improve the mobility of the doctor, the global system for mobile (GSM) communication mobile telephony network was used for connecting the server [20]. In [21], Hung and Zhang implemented a wireless application protocol (WAP) based telemonitoring system. It utilized WAP devices as mobile access terminals and allowed doctors to browse the monitored data on WAP devices in store-and-forward mode [22]. In such systems the improvement on the mobility of the patient is much less, compared to the doctor. In many previous telemedicine systems, the sensor unit consisted of an ECG data acquisition circuit, an A/D converter, and a storage unit. To provide a very limited mobility of the patient, this unit was equipped with an indoor, wireless transmitter for feeding the monitored data to a network connected PC [18, 21]. A GSM modem was equipped with a PC for real-time transmission of ECG data from a moving ambulance vehicle in [23]. In [24], Rasid and Woodward suggested a mobile telemonitoring system using a Bluetooth enabled processor unit, which transmits the monitored data to a Bluetooth mobile phone and subsequently via the GSM/GPRS (general packet radio services) network to the server. On the other hand, Engin et al. [25] used a mobile phone to transmit the measured ECG signal in real-time mode. In these designs [24, 25], the mobility of the patient is improved. However, the analysis of ECG is not performed in the place where the ECG is acquired; for example, the ECG is analyzed at the server end. In fact, there is a loss of efficiency in the use of the GSM/GPRS network because normal ECGs are also transferred, which implies a high cost. Lin et al. [26] developed a mobile patient monitoring system that integrates PDA technology and wireless local area network (WLAN) technology to transmit a patient's vital signs in real-time to a remote central management unit. The system was based on a small-sized mobile ECG recording device which sends measurement data wirelessly to the mobile phone [27]. In the mobile phone, the received data is analyzed and in cases of any abnormalities found among parts of the measurement data, it will be sent to a server. However, because of the limits of processing units within the mobile phone, the overall performance was hardly operated in an ideal condition [28]. Delay in the data transmission might also disrupt the data analysis and measurement. According to the discussed components of the telemedicine system, all systems developed can be categorized by several aspects: type of sensors, type of connection between sensors, monitoring/processing device, data communication technology, and signal processing algorithms.  Table 1 summarizes a set of telemedicine studies in the last decades along with aspects which each study concern.

In this paper, we propose a wireless telemedicine system which integrates sensor unit, processing unit, and communication unit in one chip bounded to patient's body called mobile-care unit. This will improve patient's mobility and will not affect active daily life during monitoring. To lower the cost of using GPRS network, only abnormal readings are transmitted so the proposed system operates in two modes, store-and-forward mode and real-time mode. In store-and-forward mode the care unit records and transmits patient's vital signs to the server through the internet. When an abnormal heartbeat that the doctor concerns is detected, the care unit transmits it to the server via GPRS network in real-time. The doctor at the server side could communicate with the patient also by using SMS if necessary. The proposed system also has a friendly web-based interface for medical staff to observe immediate vital signs for remote treatment which will give more mobility for medical staff. The remainder of this paper is organized as follows. The system is described in Section 2. The proposed system consists of a mobile-care unit and a server. The hardware and software designs of the mobile-care unit are described in Section 2.2. The system has been implemented and tested. Finally, Section 3 contains some discussions and conclusions.

## 2. System Design

**Figure 2:**
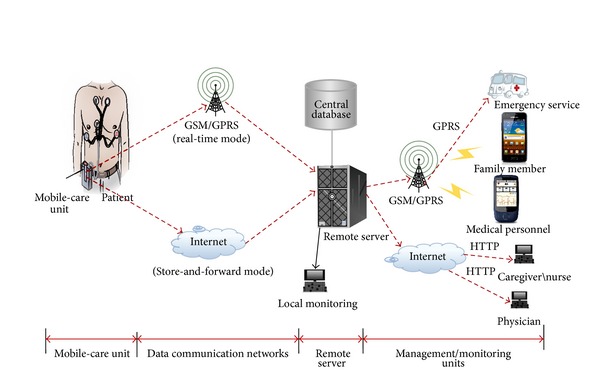
The architecture of the proposed system.

This section describes in detail the system design based on physiological sensor, signal processing, embedded system, and wireless communication and World Wide Web technologies.  Figure 2 illustrates the architecture of the proposed system.  Section 2.1 presents an overview of the system architecture. Section 2.2 describes the system components and the detail of the system operation.

### 2.1. System Architecture

The aim of this study is to design and implement a telemedicine system with intelligent data analysis based on physiological sensors, embedded system, wireless communication, and World Wide Web for vital signs monitoring, patient diagnosis, and home care. Architecture of the proposed system is shown in Figure 2. It mainly comprises the following parts.

1. Mobile-care unit: it could be bound to patient's body and could acquire real-time or periodical vital signs information without affecting their normal activities. Then an intelligent data analysis scheme is applied to identify abnormal pulses and transmits these data to the remote server by wireless communication through either internet in store-and-forward mode for normal case or cellular networks in real-time mode for abnormal case. The transmission of patient data in real-time mode can also be operated manually. Whenever the user feels uncomfortable, he can transfer his current vital signs to the management unit for advice or a checkup. By this way, the cost for using the GPRS network is lowered because only abnormal signals are transmitted. For possible long-term store-and-forward mode, the raw data can be stored in the extended secure digital flash memory contained in the mobile-care unit.
2. The remote server: it stores the received vital signs in a human physiology database and displays the physiology signals to the medical personnel through application program for diagnosis. Also, it enables remote access for caregivers and physicians to obtain vital signs through web-based interface over internet to monitor these data on their pervasive devices. After examining the vital signs data, the doctor can send a feedback MMS message to the user. The message may contain medical advice and/or a list of control commands to the mobile-care device for resending the abnormal case's vital signs data. Also remote server may alarm family member in abnormal case and call emergency service to transport patient to nearest medical center.
3. Pervasive devices: pervasive devices include laptop, personal digital assistant (PDA), and mobile phone. Through these terminal devices family members or doctors can acquire abundant information about the healthcare recipients anywhere and at any time.

### 2.2. System Components

This section details the system components of the proposed emergency telemedicine system for patient monitoring and diagnosis.

#### 2.2.1. Mobile-Care Unit

**Figure 3:**
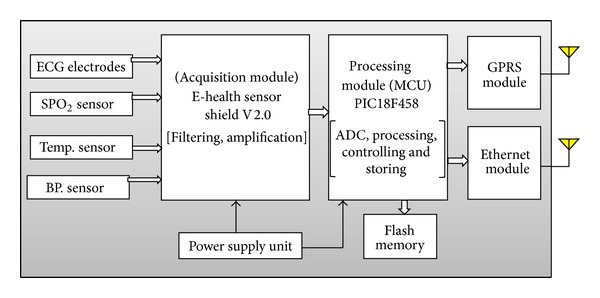
Mobile-care unit.

In the proposed system the mobile-care unit was designed to be portable and lightweight which means it is easy to carry and easy to use making patients do nothing. The mobile-care unit consists mainly of three modules. These are mainly vital-sign signals acquisition module, data control and processing module (MCU), and data communication module. Thus it can collect critical biosignals, including three-lead ECG, HR, blood pressure, and SpO2 which are vital signs. Also, it may evaluate patient status and trends in patient's medical condition and it may generate emergency alert if the patient's condition is critical. Moreover, it should support wireless communication and be compatible with global positioning information system to locate the patient position for emergency help.  Figure 3 illustrates a block diagram of mobile-care unit. Also mobile-care unit includes local data storage which is used for raw data recording together with signals processing results.

*(1) Vital-Sign Signals Acquisition Module*. Vital-sign signals acquisition module is responsible for collecting vital signs and then sends it to processing module for ADC, processing, and abnormal detection. E-health sensor shield V2.0 is selected to work as vital-sign signals acquisition module. This module can continuously acquire physiological signs like ECG, SpO2, body temperature, and blood pressure as shown in Figure 3. All of vital signs measurements will be noninvasive measurement. Noninvasive measurement of vital signs certainly has an advantage over its invasive counterpart due to the ease of use and lack of risks involved in such measurements.

*ECG Sensor*. An ECG is a bioelectric signal which records the heart's electrical activity versus time. The electrocardiogram is obtained by measuring electrical potential between two points of the body using specific conditioning circuit. In the proposed mobile-care unit ECG signals from the electrodes are amplified with a gain of 300 and filtered with the cut-off frequencies of 0.5 Hz in the high pass filter and 100 Hz in the low pass filter.

**Figure 4:**
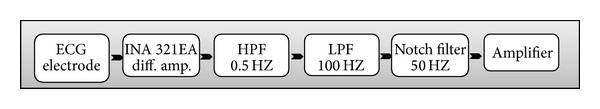
Block diagram of ECG acquisition hardware.

The ECG signals are typically 1mV peak-to-peak; an amplification of 300 is necessary to render this signal usable for heart rate detection and realizing a clean morphological reproduction. A differential amplifier with gain of 20 avoids the noises overriding the ECG signals; this is achieved by an instrumentation amplifier (INA321EA), CMRR of 100 dB, and at the end an operational amplifier (Analog AD8625) is used to amplify the signal with a gain of 15. The ECG signals are restricted in bandwidth of 0.5–100 Hz using second order Butterworth high pass and low pass filters after the first stages of amplification. The power line interference in the ECG signal is filtered by a 50 Hz notch filter, which is user selectable to avoid loss of 50 Hz component of the ECG signals. Then the ECG signal is fed to the analog input of processing unit for digitizing and analysis.  Figure 4 illustrates the block diagram of ECG signal acquisition hardware.

**Figure 5:**
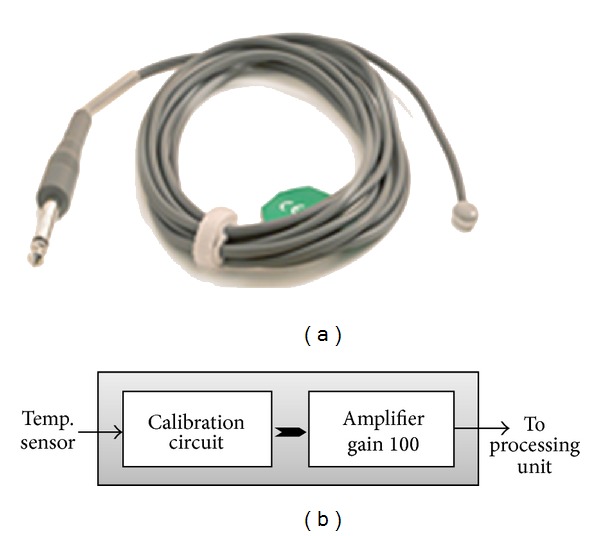
(a) Temperature sensor. (b) Signal conditioning circuit.

*Temperature Sensor.* The temperature of a healthy person is about 37°C; it may slightly or temporarily increase in hot environment or in physical activity; in extreme effort, the increase may be very high. It is of great medical importance to measure body temperature. The reason is that a number of diseases are accompanied by characteristic changes in body temperature. Likewise, the course of certain diseases can be monitored by measuring body temperature, and the efficiency of a treatment initiated can be evaluated by the physician. An industrial CMOS integrated-circuit temperature sensor shown in Figure 5(a) was chosen and connected to signal conditioning circuit shown in Figure 5(b) to calibrate and amplify the signal before feeding it to processing unit.

**Figure 6:**
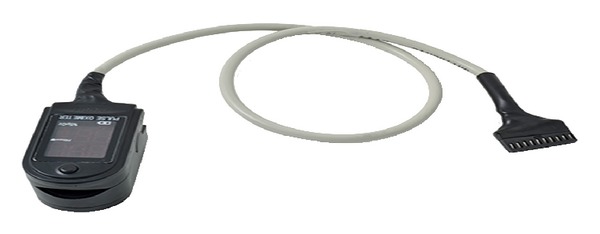
SpO2/HR sensor.

*Blood Oxygenation Measurement (SpO2) and Heart Rate.* SpO2 or pulse oximetry is the measure of oxygen saturation in the blood, which is related to the heart pulse when the blood is pumped from the heart to other parts of the human body. When the heart pumps and relaxes, there will be a differential in absorption of light at a thin point of a human body. Oxygenated hemoglobin absorbs more infrared light waves and allows more red light waves to pass through. However, deoxygenated (or reduced) hemoglobin absorbs more red light waves and allows more infrared light waves to pass through. This unique property of hemoglobin with respect to red and infrared light wave allows oxygen saturation to be detected noninvasively. Pulse oximetry is a simple yet reliable method to measure oxygen saturation that otherwise would have to be measured by invasive methods. Red (660 nm) and infrared (940 nm) LEDs were chosen and populated onto a custom-made sensor shown in Figure 6. Besides oxygen saturation in the blood the used sensor also provides heart rate. The output of the SpO2/HR sensor is fed to processing unit through acquisition module. Specifications of various physiological parameters monitored in the proposed system are listed in Table 2.

**Figure 7:**
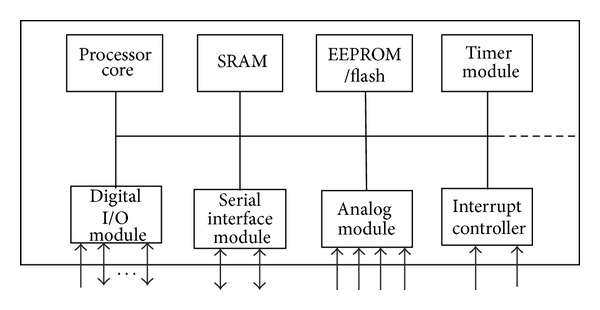
Architecture of microcontroller.

*(2) Data Control and Processing Module.* Data control and processing module is the heart of the medical care unit. The main function of this module can be divided into two parts: in the first part the developed algorithm synchronizes, controls, and maintains the accurate operation and communication of all the other modules. In the second part the developed algorithm digitizes and processes the acquired vital-sign signals to determine if their respective values are above the preset limit or not. If any or all of these values are above their respective critical values then triggering alarm is made. After that all processed data is transmitted to communication layer. This module mainly consists of a microcontroller which is chosen to verify certain specifications. Microcontrol unit (MCU) with powerful processing and control capability is needed to adapt a large amount of data acquiring and processing. Moreover, this module also possesses a high degree of system integration as well as more extension interfaces. We select 8 bit PIC18F458 microcontroller as the MCU of medical care unit. It has input-output circuitry and peripherals built-in, allowing it to interface more or less directly with real-world devices such as sensors. Modern microcontrollers often need little external circuitry. Among the most accessible are the PIC microcontrollers. A microcontroller already contains all components which allow it to operate stand alone and it has been designed in particular for monitoring and/or control tasks. In consequence, in addition to the processor it includes RAM, ROM, and EEPROM memory units, SPI, I2C, CAN, ADC, and UASRT interface controllers, one or more timers, an interrupt controller, and general purpose I/O pins.  Figure 7 shows the architecture of the MCU.

We can summarize the main functions of MCU in the proposed system as follows.

**Figure 8:**
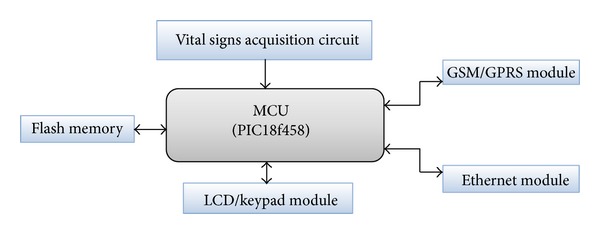
Functions of MCU.

1. It receives and digitizes the signals acquired from vital sign sensors.
2. It controls the operation of all connected modules as shown in Figure 8.
3. It processes the received signals using different sorts of processing techniques and algorithms.
4. It sets up a connection with the remote server and transmits to it the analysis results and raw data using communication techniques.
5. It stores analysis results and raw data to flash memory.

**Figure 9:**
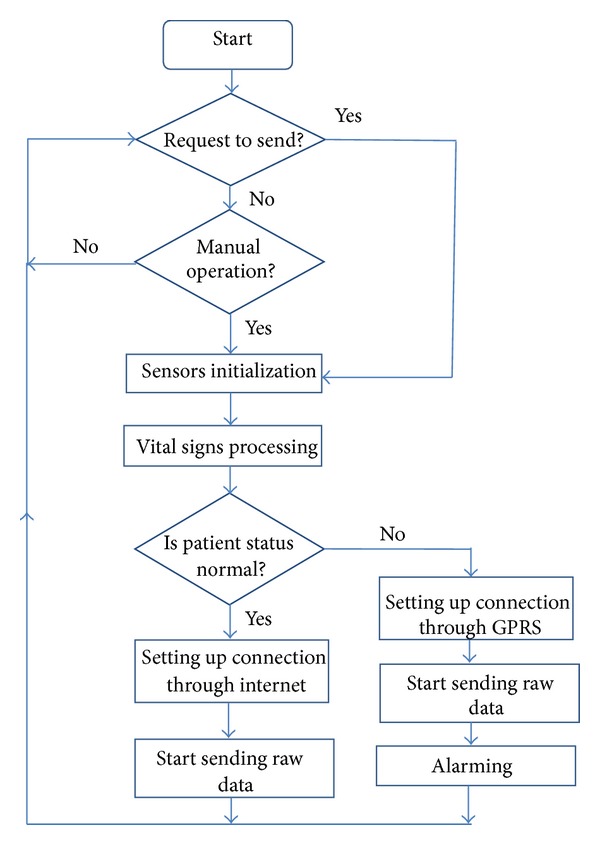
Work flow about mobile-care unit.

*(3) Software Components of the Processing Unit.* The MCU controls and coordinates all activities of mobile-care unit.  Figure 9 shows the workflow about the mobile-care unit. Software has been written in C language to simulate MCU and its components. It is based on the following concepts.

1. Sensor and module initialization component: it is in charge of starting, initializing, and configuring the medical care unit.
2. Vital signs perception component: it acquires the values of vital signs from sensor nodes.
3. Vital signs processing component: it realizes data conversion and processing and carries out patient diagnosis by determining the health status of patient.
4. Information transmission component: data exchange between mobile-care unit and server is realized with the help of this component.
5. Information receiving component: it helps the node to receive the controlling or inquiring requests from the server.
6. Exception notification component: when the abnormal sensing information appears, it sends a message to the server immediately and sends out the alarm as soon as possible.

**Figure 10:**
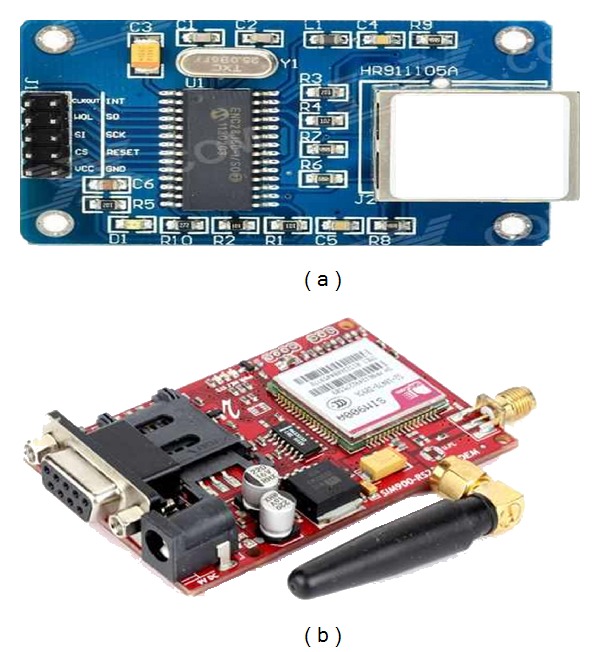
(a) ENC28J60 Ethernet module and (b) Sim900 GSM/GPRS module.

*(4) Data Communication Module.* Data communication module helps the medical staff to get patient's physiological data by connecting medical care unit to other networks such as cellular network or internet. It is responsible for uploading the received vital signs data to the remote care server through cellular network to carry out the patient‘s health condition monitoring and diagnosis. This module operates in two modes: store-and-forward mode in which mobile-care device records patient's vital signs continuously up to specified period and transmits it to the remote server and real-time mode which operates when an abnormal heartbeat is detected. Mobile-care unit transmits all vital signs to the remote server via GSM/GPRS network in real-time. In the proposed system, medical care unit can send data through internet network either by UDP or TCP protocols using ENC28J60 Ethernet module shown in Figure 10(a). For real-time connection in emergency cases vital signs are transmitted through GSM/GPRS networks using sim900 GSM/GPRS module shown in Figure 10(b). The GSM/GPRS module used operates at Quad-Band 850/900/1800/1900 MHz and is controlled via AT commands.

#### 2.2.2. Remote Server Unit

In the application of telemedicine, the medical information usually needs to be distributed among medical doctors and display, archival, and analysis devices. Therefore, the remote server unit is developed with the purpose of receiving, storing, and distributing the vital sign data from patients. The server is composed of presentation tier, web tier, and database tier. A multitier architecture allows for separation of concerns where any tier in the system can be expanded and updated with minimal or no effect on the client tiers. The following subsections discuss the three tiers further.

*(1) Presentation Tier.* The presentation tier allows the authorized user to interact with the received patient's data through application program developed using C# language. The interface design provides most of the general as well as functional requirements as follows.

- Access constraints are applied all the time based on the authorized user registered in the database.
- It includes lists of patients and personal information about patients.
- It displays patients' vital signals and sets thresholds for each measurable parameter.
- It alerts healthcare providers in abnormal cases.
- It adds new patient, new consultation, and drug prescription.
- It shows past medical records for all patients including diseases, past surgeries, clinical findings, past medication, allergies, and images.
- It provides search for all registered patients by patient's ID or patient's name.
- It shows notations (patient experience) while taking measurements.
- It sends messages including instructions for patients and drug prescription.
- Sensor data will be automatically reloaded at predefined time intervals to keep the view updated.

**Figure 11:**
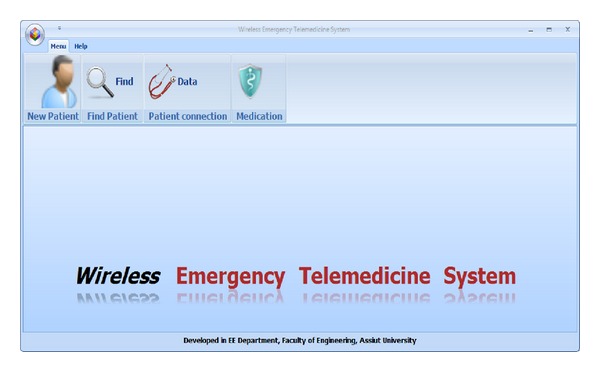
Screen shot of the developed interface main page.

**Figure 12:**
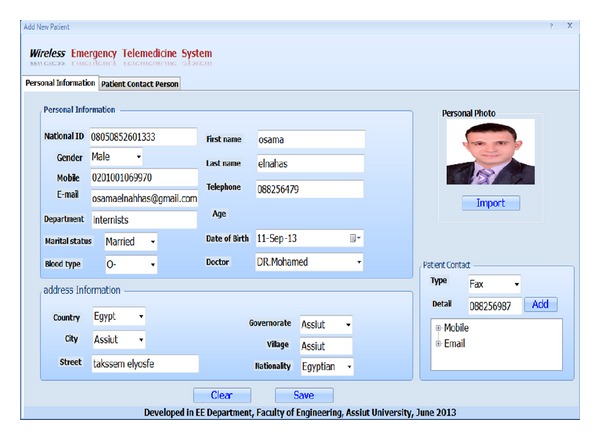
Add new patient screen shot.

**Figure 13:**
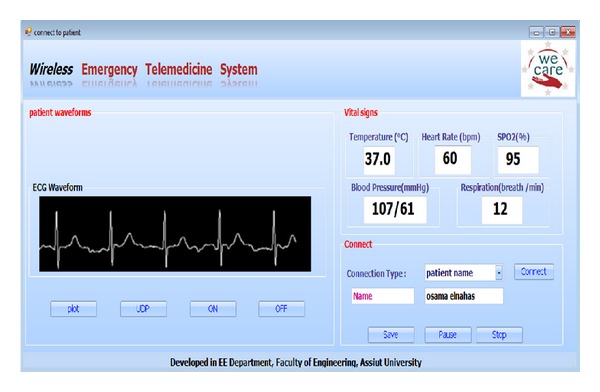
Displaying patient's vital signs.

Screen shots of the developed software are shown in Figures 11, 12 and 13.

**Figure 14:**
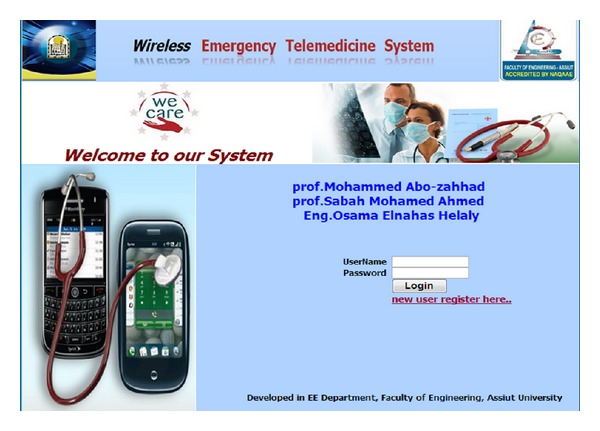
User authentication page.

**Figure 15:**
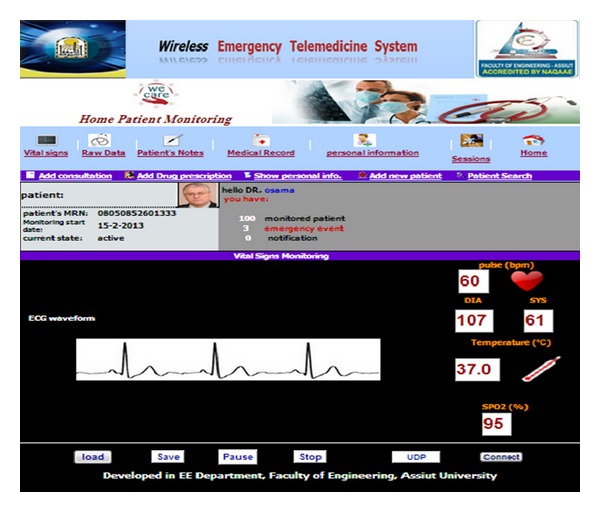
Displaying patient's vital signs.

*(2) Web Tier.* Web tier allows different users such as physicians, doctors, and medical center to interact with the server through a web interface. Remote web user will have real-time and continuous access to patients' vital signs through the internet. The web user interfaces with the web components using HTTP protocol over TCP/IP connection. The information and content are presented to the user using an internet browser through webpage designed using Microsoft visual studio 2010. The designed webpage provides the most general functions of developed application in the presentation tier discussed previously. Screen shots of the designed webpage are shown in Figures 14 and 15.

*(3) Database Tier.* The database tier is responsible for storage, retrieval, update, and integrity of the data to and from the presentation and web tiers. The most common way to access the database is by using drivers that allow accessing a relational database management (RDBMS) to query or update the data records. The driver used in the implementation of the proposed system is JDBC drivers and the database is deployed on a SQL database server. The database tier provides the ability to do the following:

- store, retrieve, and update patient's record including his/her medical personnel's contact information and other details;
- store and retrieve the received physiological sensor data transmitted by medical care unit;
- store, retrieve, and update patient's consultations and drug prescriptions;
- store and retrieve patient's notation during sessions;
- store, retrieve, and update registered doctors, physicians, and nurses;
- store, retrieve, and update the ECG data, record time, location of the R wave, and estimated ECG beat type.

**Figure 16:**
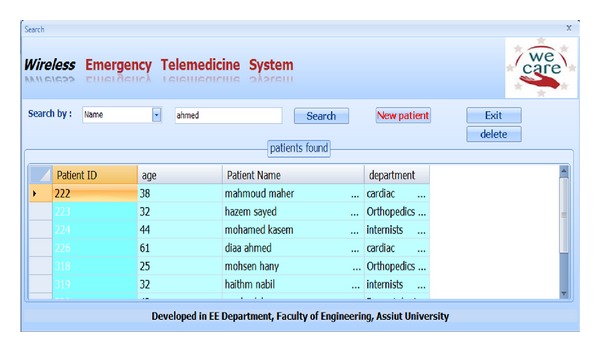
Screen shot for how to search.

Figure 16 shows screen shot for how to search.

#### 2.2.3. Monitoring Units

Web tier in the remote server is designed to allow remote user to acquire abundant information about the healthcare recipients anywhere and at any time using pervasive devices such as laptop, PDA, and mobile phone. Finally we can say that the proposed system can operate in the following three situations.

1. Time-based connection: all data needed by the remote caregivers or specialists should be uploaded. Data compression is essential to limit the upload time. In this situation the remote caregiver should determine time schedule for uploading all patient data to remote server. The time schedule is stored in the mobile-care unit so it will upload data according to this time schedule.
2. Emergency connection: to lower the cost of using GSM/GPRS network we develop algorithm which detects abnormal heartbeats. So during sensor monitoring, if the mobile-care unit detects an abnormal condition it sends the collected data to the remote server in order to receive clinical assessment and treatment planning.
3. (Event awareness) connection on demand: the mobile-care unit uploads the amount of data requested by the remote caregivers or specialists to monitor the health status of the patient.

## 3. Conclusion and Future Scope

This paper proposes the design and implementation of a wireless telemedicine system, in which all physiological vital signs are transmitted to remote medical server through both cellular networks in emergency case and internet in normal case for long-term monitoring. By this, the cost of using GSM/GPRS network is reduced as only abnormal cases will be transmitted through cellular network. Also the proposed system presents friendly web-based interface for medical staff to observe immediate vital signs for remote treatment. Comparing this system with other systems which are mentioned in the introduction [18–28], the proposed system integrates sensor unit, processing unit, and communication unit in one chip bound to patient's body called mobile-care unit, so patient could do his/her daily activities during monitoring. In other words, this will improve the mobility of patient. Also the proposed system provides an ability to continuously monitor patient's vital health conditions instead of the discrete measurements.

In the future, a lot of work could be done in the three main aspects of telemedicine systems to enhance the healthcare services. The three main aspects are type of sensors, signal processing algorithms, and data communication technology. In the sensor layer wireless sensor network of wearable noninvasive sensor units can be designed. Fabrication of sensors can be improved to obtain small size and low power sensors to improve patient's mobility and prolong network lifetime. Also we can increase the number of transmitted vital signs to have a complete picture of patient's case. For more improvement in telemedicine systems, many medical algorithms can be developed to help in patient diagnosis and early detection of cardiovascular diseases and real-time analysis of vital signs can be performed in the place where the vital signs are acquired. The latest achievement on a smart phone market provided an opportunity to integrate smart phones in telemedicine systems. For example, android based mobile phones patient monitoring application could be developed which allows doctors to monitor the health status of a patient using the easy to understand user interface (UI). This application also provides alerts, reminders, and emergency notifications for vital measurements to help doctors to take timely decisions in emergency situations. Finally for data communication technologies, in many countries, 3G mobile networks like the UMTS are currently installed and operating, which provide bandwidth up to 2 Mbps maximum (typically hundreds of kbps) [30]. This will enable the transmission of more information like continuous 12 leads of ECG when monitoring cardiac patients from a moving ambulance vehicle. Furthermore, the current introduction of new services like video telephony through wireless networks is an addition that can help with communications between a healthcare provider (nurse, paramedics) and an expert doctor. The current activities in what is termed as the 4G mobile networks promise ubiquitous access to differing radio network technologies, thus offering, beyond extended coverage, also the most effective connection mode at the point of contact, even using simultaneously more than one wireless access technologies and seamlessly moving between them. The use of locating systems such as the global positioning system (GPS), the geographical information systems (GIS), and intelligent traffic control systems also has the potential to improve healthcare services, for example, when a moving ambulance vehicle is trying to reach a patient using the fastest route or when an ambulance vehicle carrying a patient is trying to get to the base hospital.

## Figures and Tables

**Table 1:** Set of telemedicine studies along with aspects which each study concerns.

Reference number	Biosignal sensors	Communication technology		Medical algorithm	Comments
		GSM/GPRS	Internet		
[29]	ECG, BP, HR TEMP.	*√*	*√*		WSN, type of localization method for patients and an energy efficient transmission strategy, video streaming.

[3]	HR, SPO2, TEMP., RESP.	*√*			Implement a prototype of telemedicine system based on wireless technology using GSM and GPS.

[4]	Weight, activity, BP	*√*	*√*		Android application for monitoring and using Bluetooth enabled sensors.

[5]	BP, HR, TEMP.	*√*	*√*		Design of sensors to reduce power consumption using VLSI and FPGA.

[6]	ECG, HR, SPO2, TEMP., RESP.		*√*		Wearable belt; high quality and flexible modules for signal conditioning are designed and assembled together.

[9]	ECG, BP, HR TEMP., PPG		*√*	*√*	Small rang RF transmission, smart wearable vest, deriving BP and HR from ECG.

[14]	ECG		*√*	*√*	QRS detection algorithm, extraction of heart rate variability, implemented in the PDA and GPS.

[13]	ECG	*√*	*√*	*√*	A real-time ECG classification algorithm, GPS, and a real-time R wave detection algorithm.

[15]	Pulse signal		*√*	*√*	Intelligent data analysis scheme to diagnose abnormal pulses for exploring potential chronic diseases.

[22]	ECG, HR, SPO2, TEMP., RESP.		*√*		Vital signals are acquired from the monitor using the RS232 interface and transmitted through the internet.

[23]	ECG, BP, HR TEMP.	*√*	*√*	*√*	Commercial monitors are used for the acquisition of biosignals and Huffman algorithm for ECG signal compression, GSM, GPRS, POTS, or satellite.

**Table 2:** Specification of various physiological parameters monitored.

Physiological parameter	Specifications	Typical values for average healthy person
ECG	Frequency: 0.5 HZ–100 HZ Amplitude: 0.25–100 mv	R-WAVE amplitude: >4.5 mv QRS complex: (0.04–0.12) msec
Heart rate (HR)	40–220 beats per minute	60–100 beats/minute
Body temperature	32°C–40°C	About 37.5°C
Blood pressure	Systolic: 50–300 mmHg Diastolic: 40–140 mmHg	Systolic: less than 120 mmHg Diastolic: less than 80 mmHg
Blood oxygenation (SpO2)	Measurement range: 70–100%	Around 94% to 99%
Respiratory rate	2–50 breath/min.	Adults: 12–24 breaths per minute
